# The contribution of hypochondria resulting from Corona virus on the occupational productivity loss through increased job stress and decreased resilience in the central workshop of an oil refinery: A path analysis

**DOI:** 10.1016/j.heliyon.2021.e06808

**Published:** 2021-04-18

**Authors:** Saeid Yazdanirad, Marzieh Sadeghian, Mahsa Jahadi Naeini, Milad Abbasi, Seyed Mahdi Mousavi

**Affiliations:** a School of Health, Shahrekord University of Medical Sciences, Shahrekord, Iran; b Department of Occupational Health Engineering, School of Public Health, Ahvaz Jundishapur University of Medical Sciences, Ahvaz, Iran; c Department of Occupational Health Engineering, School of Public Health, Isfahan University of Medical Sciences, Isfahan, Iran; d Department of Occupational Health Engineering, School of Public Health, Tehran University of Medical Sciences, Tehran, Iran

**Keywords:** Hypochondria, Corona virus, Occupational productivity, Job stress, Resilience

## Abstract

The prevalence of contagious viral-infectious diseases such as COVID19 cause the economic problems in addition to harmful effect on the people health. The present study was aimed to determine the contribution of hypochondria resulting from Corona virus on the occupational productivity loss through increased job stress and decreased resilience in the central workshop of an oil refinery. This cross-sectional study was conducted on 275 subjects in the spring of 2020 in one of the oil and gas industries in southern Iran. To collect the data, the demographic, standard hypochondria, CD-RSC resilience, job stress, productivity questionnaires were sent electronically along with a guide to completing them, as well as study objectives. People were given two weeks to complete the questionnaires and send them electronically to the research team. Participation rate was 80%. Finally, a model based on the defaults was developed in AMOS software and the relationships between the variables were examined. The results showed that corona hypochondria could affect productivity in two ways. In the first place, hypochondria significantly increases job stress, thereby reducing productivity. The indirect effect of hypochondria on productivity in this direction was -0.09. In another way, hypochondria significantly reduces resilience and thus lowers productivity. The effect of hypochondria on productivity was equal to -0.04. Based on the results, the fit of the drawn model was confirmed. The results of the study generally suggested that coronavirus disease has caused the spread of hypochondria mental disorder. Hypochondria could reduce the productivity of workers through two ways of increasing job stress and reducing workers' resilience.

## 1 Introduction

Emerging contagious viral-infectious diseases are a major challenge in the 21st century. In recent years, the prevalence of diseases such as Ebola and Middle East Respiratory Syndrome has caused great damage to communities in terms of health and the economy (Anderson et al., 2020; Decaro and Lorusso, 2020). According to the World Health Organization, Coronavirus Disease 2019 began on December 30, 2019 in Wuhan, China, and quickly affected many people in the country (Pan et al., 2020). Since the coronavirus is transmitted through the respiratory tract, the virus affected almost all countries in less than one month (Wu and McGoogan, 2020). To prevent the spread of the virus, the involved countries asked all people to quarantine themselves at home and follow health protocols (Khan et al., 2020a, 2020b). Thus, Fear of illness, interference with daily activities, reduced social relationships, job and financial problems, and other consequences in these situations threaten society's mental health (Gangopadhyaya and Garrett, 2020; Pancani et al., 2020; Sloan et al., 2020). One of the psychological consequences of COVID19 outbreak is hypochondria disorder or persistent fear of having a serious illness (Wang et al., 2020b). Other diseases also may create this disorder. Statistics show that 4–5% of patients in public medical offices have hypochondria (Leibbrand et al., 2000). However, coronavirus disease is more likely to cause higher rates of hypochondria due to its high prevalence and unknown dimensions. Coronavirus transmission capabilities from person to person, possible genetic mutations that increase the pathogenicity of the virus, or the lack of vaccines and definitive treatment for the virus are all issues that raise many concerns about the virus and may contribute to hypochondria (Lee and Crunk, 2020).

The oil and gas refineries are one of the most important industries in developing countries and play a vital role in their economies. Therefore, productivity in these industries is very substantial (Mojarad et al., 2018). One of the characteristics of a healthy organization is that the physical and mental health of employees is considered as important as its production and benefit. It seems that hypochondria can affect the productivity of the workers and the whole organization (Quick, 1990). The effect of hypochondria on people's productivity may be moderated by the two variables of job stress and resilience. The workers occupied in the oil and gas industries expose to various risk factors such as harmful agents, shift work, uniform conditions, and job insecurity, which can cause job stress (Habibi et al., 2014). The results of a study showed that the prevalence of job stress in an Iranian oil company was equal to 43% (Shojaei et al., 2020). The hypochondria can probably increase stress in the workers of these industries because of worry on health. Moreover, the hypochondria maybe can decrease the people ability for withstanding the stress and mental load, which named as resilience. The studies have shown that 75% of medical problems are directly related to stress, so stress is responsible for significant costs associated with health care, poor performance, and low productivity (Birmes et al., 2005; Lu et al., 2019) Stress has negative consequences, including manpower relocation, absenteeism, reduced quality, reduced productivity, and reduced job satisfaction (Biganeh et al., 2021; Mullen et al., 2018). Given that the most important effective factor in achieving high productivity is efficient human resources, the stressful conditions can reduce the productivity of the oil and gas industries.

Another important variable is resilience, which can moderate the effect of hypochondria on productivity. Resilience is defined as the capacity of individuals to withstand adversity and to continue to grow individually in times of crisis. Also, in another definition, resilience is often referred to as the ability to dominate or return to after experiencing stress and severe challenge (Joseph and McGregor, 2020; Shakir et al., 2020). Hypochondria may also reduce people's resilience. Factors affecting resilience are divided into four categories based on previous studies, including individual factors, environmental and organizational factors, reaction to new situations, and educational interventions (Huey and Palaganas, 2020). It has been reported that people with low resilience are prone to stress, anxiety, and interpersonal problems when they are faced to life problems, and may exhibit risky behaviors (Laird et al., 2019). Having these characteristics can protect a person against crises such as COVID 19 disease, which has caused much stress in the majority of people of society. Given these relationships have not investigated so far, as well as the importance of the employee's productivity in the oil and gas industries in the conditions of widespread prevalence of COVID 19, the present study was aimed to determine the contribution of hypochondria resulting from Corona virus on the occupational productivity loss through increased job stress and decreased resilience in an oil refinery. In this study, it was assumed that the hypochondria due to COVID 19 can increase the job stress and decrease the resilience and cause decreased productivity through these ways.

## 2 Materials and method

### 2.1 Participants

A cross-sectional study was conducted in the spring of 2020 in an oil refinery in southern Iran. The population of this study was the technical workers of the central workshop in this industry. This place was selected because there are persons with various occupations such as turning, welding, metalworking, tool and machine repairing, and industrial washing in this part. Also, all of the employees work in one industrial hall and there were no separate sections by the wall, and there are many interactions between them. A large number of employees in each shift, the shared use of hand tools, workstations with the short distance from each other, and low use of respiratory masks because of high ambient temperature in the central workshop provide the conditions to create the COVID19-related fear and hypochondria. Moreover, the central workshop is one of the main parts of the oil and gas industries and productivity has high importance in it. Participants were selected using a simple random sampling method based on inclusion criteria. Inclusion criteria included: more than one year of work experience, no active corona, no chronic diseases such as cancer, diabetes, AIDS, cardiovascular diseases and MS, no mental disorders, no mental medication, and no addiction. The exclusion criteria were: lack of willingness to participate in the study, lack of cooperation to complete the questionnaires, and completing the questionnaires randomly and inappropriately. Initially, a list of names of employed people was prepared and 500 people were randomly selected. Then, the medical records of these people were studied and those who did not have the criteria to enter the study were excluded from the study. Finally, 330 people remained in the study. The percentage of answers to the questionnaires was 80%, and a total of 275 people completed and sent the questionnaire.

### 2.2 Data collection

The protocol for implementation of this study was reviewed and approved by the Medical Ethics Committee of Ahvaz Jundishapur University of Medical Sciences. All steps of the study were accordance with the ethical code IR. AJUMS.REC.1399.634. To collect the data, the selected individuals were first contacted by telephone, the steps and objectives of the study were explained to them, and they were invited to participate in the study. Those who agreed were given an e-mail or social networks address, and questionnaires were sent electronically along with a guide to completing them, as well as study objectives. Moreover, all participants were asked to fill out the consent form developed by the medical ethics committee, and written informed consent was obtained from all of them. The e-mail and also mentioned a phone number for contacting the research team as a way to communicate about possible problems when completing the questionnaires. People were given two weeks to complete the questionnaires and send them electronically to the research team. After the deadline, those who had not completed the call were contacted, and those who did not wish to continue their study were excluded. Finally, 275 people completed the questionnaires completely.

### 2.3 Tools

In the present study, several questionnaires were used to collect data, including demographic questionnaire, standard hypochondria questionnaire, resilience questionnaire, job stress questionnaire, and productivity questionnaire, which will be mentioned below. All used questionnaires, except for the demographic questionnaire, have been fully adopted from the questionnaires that previous researchers have produced and their validity and reliability have been evaluated by them.

### 2.4 Demographic questionnaire

The questionnaire included general data such as age, work history, occupation, place of work, level of education, shift work type (fixed shift or rotating shift), and history of corona in the person or his/her family.

### 2.5 Standard hypochondria questionnaire

The hypochondria questionnaire was designed and developed in 1980 by Evans to familiarize with hypochondria. The questionnaire has 36 questions, and based on the Likert scale, it measures hypochondria by asking questions such as (how much do you think you are exposed to different diseases compared to your age group?). This questionnaire contains 36 questions and people based on the score obtained are placed in healthy groups (score 0–20), borderline (21–30), mild (31–40), moderate (41–60) and severe (above 60) (Evans, 1980). The validity and reliability of the Persian version of this questionnaire have been evaluated in the study by Khani et al. and Cronbach's alpha coefficient reported for it has been 0.86 (Khani et al., 2016) In addition, the high correlation of the questionnaire with other measurement tests of hypochondria such as SCL-90 indicates its proper validity.

### 2.6 CD-RSC resilience questionnaire

The questionnaire consists of 25 phrases designed in 2003 by Kerner and Davison. The scoring is based on Likert scale (totally false score 0, rarely score 1, sometimes true score 2, often true score 3, usually true score 4). Thus, the minimum acquired score is 0 and the maximum is 100. The cut-off point of this questionnaire is 50 points and the higher the score, the higher the resilience. The validity of the questionnaire was reported by Kerner and Davison as 0.89 (Connor and Davidson, 2003). The validity of the Persian version of the resilience questionnaire by Samani and et al. was equal to 0.87 and was confirmed (Samani et al., 2007).

### 2.7 Job stress questionnaire

The questionnaire consists of 60 questions that assess 6 dimensions including role workload, role incompetence, role duality, role range, responsibility, and physical environment by ten phrase questions. The scoring of the SPIO job stress questionnaire is based on the 5-point Likert scale. For each phrase, 5 options are considered from "Never" equal to 1 point, "Sometimes" 2 points, "Often" 3 points, "Usually" 4 points, and "Always" 5 points. The range of this questionnaire is between 60 and 300, and higher scores indicate high stress levels. General stress levels are divided into four categories: low stress (score 50–99), low to moderate stress (100–149), moderate to severe stress (150–199), and severe stress (200–250) (Osipow, 1998). During the research of Sharifian et al., the validity and reliability of the Persian version of this questionnaire were measured, where its Cronbach's alpha coefficient was calculated and reported to be 0.83 (Sharifian et al., 2006).

### 2.8 Productivity questionnaire

The Standard Manpower Productivity Questionnaire was developed by Hersey and Goldsmith in 1980. This questionnaire consists of 26 items and is based on a 5-point Likert scale. The scoring method is based on the Likert spectrum: very high score of 5, high score of 4, to some extent score of 3, low score of 2, and very low score of 1 are assigned. The Persian version validity and stability of this questionnaire were studied based on Hedayati's study and Cronbach's alpha coefficient was reported to be 0.81 (Hedayati et al., 2009; Hersey and Goldsmith, 1980).

### 2.9 Data analysis

**Figure 1:**
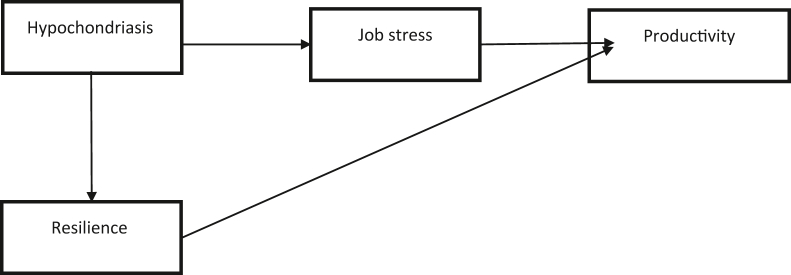
The theorical model.

Initially, the data was introduced into SPSS software version 22 for analysis. Then, the normality of the variables was examined using curvature and skew curves with the results showing that the model variables have a normal distribution. Thus, Pearson test was used to investigate the correlation between the studied variables. Finally, a model based on the defaults was developed in AMOS software and the relationships between the variables were examined. Figure 1 shows the theorical model. Absolute, comparative, and normed fitness indicators were also used to determine the fit of the model.

## 3 Results

### 3.1 Demographic characteristics of workers

The mean age and standard deviation of participants were 43.26 and 9.44, respectively. Table 1 reports the statistical distribution of other demographic characteristics of the participants. According to the results, most of the participants were over 40 years old (69.5 %), had more than 20 years of work experience (69.5 %), diploma and sub-diploma level (72.0 %) and rotating shift work (56.0 %).

**Table 1:** The statistical distribution of other demographic characteristics of the participants.

Variables		Frequency	Relative frequency
Age	Less than 30	31	11.3
	30 to 40	53	19.3
	40 to 50	100	36.4
	More than 50	91	33.1
Career length	Less than 10	31	11.3
	10 to 20	53	19.3
	20 to 30	100	36.4
	More than 30	91	33.1
Education level	Sub diploma	104	37.8
	Diploma	94	34.2
	Associate degree	77	28.0
Rotating shift work	Yes	154	56.0
	No	121	44.0

### 3.2 Statistical distribution of variables

The statistical distribution of the studied variables is shown in Table 2. The results show that the statistical distribution of the studied variables is normal. The mean prevalence of hypochondria was 33.37 and the standard deviation was 14.87. The mean total stress and resilience score was 107.4 ± 47.86 and 78.56 ± 20.95, respectively. Also, the average employee productivity score was 85.15 and the standard deviation was 32.58.

**Table 2:** The statistical distribution of the studied variables.

Variables		Range	Mean	Standard deviation
Hypochondriasis		10.00–65.00	33.75	14.87
Stress	Work load	10.00–50.00	16.98	9.71
	Incompetence of role	10.00–50.00	16.02	9.19
	Duality of role	10.00–50.00	15.65	9.55
	Range of role	10.00–50.00	15.51	8.66
	Responsibility	10.00–50.00	19.48	11.94
	Physical environment	10.00–50.00	23.09	13.02
	Total score	60.00–300.00	107.40	47.86
Resilience		25.00–100.00	78.56	20.95
Productivity		25.00–135.00	85.15	32.58

### 3.3 Correlation between variables

Table 3 outlines the correlation matrix of the variables under study. The results of Pearson correlation showed that all aspects of job stress had a significant positive correlation with hypochondria score. Meanwhile, responsibility dimension with coefficient of 0.374 showed the highest correlation with the hypochondria score. The results also showed that the dimensions of job scope, accountability, and physical environment related to job stress had a negative correlation with resilience. Meanwhile, the responsiveness dimension with coefficients of -0.189 had the highest correlation with resilience score. Based on the results, all aspects of job stress, except for workload, had a significant negative correlation with productivity. Meanwhile, the job dimension with a coefficient of -0.247 revealed the highest correlation coefficient with the productivity score. In addition, the results showed that all variables of job stress, hypochondria, resilience, and productivity had significant correlations with each other. Based on the results, job stress with a coefficient of 0.253 indicated the highest correlation with productivity score. The results also showed that hypochondria with a coefficient of 0.406 had the highest correlation with job stress.

**Table 3:** The correlation matrix of the variables under study.

Variable		1	2	3	4	5	6	7	8	9	10
1	Work load	-									
2	Incompetence of role	0.512∗∗	-								
3	Duality of role	0.567∗∗	0.552∗∗	-							
4	Range of role	0.471∗∗	0.429∗∗	0.569∗∗	-						
5	Responsibility	0.462∗∗	0.394∗∗	0.422∗∗	0.534∗∗	-					
6	Physical environment	0.509∗∗	0.394∗∗	0.389∗∗	0.544∗∗	0.727∗∗	-				
7	Total stress	0.747∗∗	0.676∗∗	0.720∗∗	0.748∗∗	0.791∗∗	0.808∗∗	-			
8	Hypochondriasis	0.308∗∗	0.237∗∗	0.279∗∗	0.272∗∗	0.374∗∗	0.327∗∗	0.406∗∗	-		
9	Resilience	0.027	−0.031	−0.043	−0.159∗∗	−0.189∗∗	−0.122∗	−0.121∗	−0.182∗∗	-	
10	Productivity	−0.082	−0.233∗∗	−0.163∗∗	-0.247∗∗	−0.233∗∗	−0.227∗∗	−0.253∗∗	−0.162∗∗	0.249∗∗	-

∗P < 0.05.

∗∗P < 0.01.

### 3.4 Path analysis

**Figure 2:**
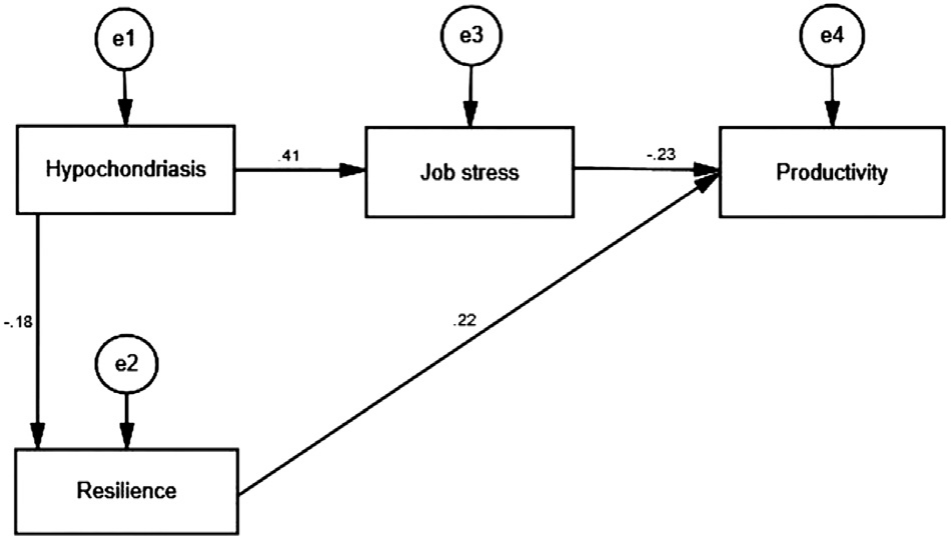
The final theoretical model proposed in the present study.

Initially, several models were drawn, and based on the indices of model fit and the proposed relationships, the original model was modified. The final model proposed in the present study is shown in Figure 2. In Figure 2, all relationships were significant. The results showed that corona hypochondria could affect productivity in two ways. In the first place, hypochondria significantly increases job stress, thereby reducing productivity. The indirect effect of hypochondria on productivity in this direction was -0.09. In another way, hypochondria significantly reduces resilience and thus lowers productivity. The effect of hypochondria on productivity was equal to -0.04. Table 4 also shows the fit indices of the analyzed model. Based on the results, the fit of the drawn model was confirmed.

**Table 4:** The fit indices of the analyzed model.

Indices	Name	Fitness	Obtained value
Absolute fitness indices	Goodness-of-fit index (GFI)	>0.9	0.999
	Adjusted goodness-of-fit index (AGFI)	>0.9	0.999
Comparative fitness indices	Normed fit index (NFI)	>0.9	0.998
	Comparative fit index (CFI)	>0.9	0.999
	Incremental fit index (IFI)	0–1	0.999
Normed fit index	Root mean squared error of approximation (RMSEA)	<0.1	0.010
	Normed Chi-square (X2/df)	1–3	1.076
P value		>0.05	0.584

## 4 Discussion

### 4.1 Statistical distribution of variables

In this study, the effect of hypochondria disease caused by COVID 19 was discussed on productivity with the mediating role of job stress and resilience in the central workshop of an oil refinery. A total of 275 employees working in the central workshop with the age range of 25–55 years formed the statistical population of this study. The results of the present study showed that the values of hypochondria, stress, resilience, and productivity variables were in the ranges of borderline to moderate, low to severe, moderate to high, and low to high. The central workshop has different parts such as turning, welding, metalworking, tools and machine repairing, and industrial washing. all equipment in the operational units is sent to this part for constructing or repairing. The current job procedures include the initial visit of the workplace, issuance of work permits, implementation of work, and delivery of work. A team is usually done its tasks under the supervision of a safety officer. With the onset of the corona epidemic, preventive measures and health protocols such as education on it, disinfection of work surfaces, and distribution of personal protective equipment were performed to maintain the staff's health and reduce the infection risk. However, a large number of employees in each shift, the shared use of hand tools, workstations with the short distance from each other, and low use of respiratory masks because of unfavorable weather conditions in the central workshop have been reduced the efficiency of the preventive protocols. Therefore, this unsafe situation could provide the conditions to make the COVID19-related fear, hypochondria, stress, and other consequences.

### 4.2 Correlation between variables

The results of the present study generally showed that all aspects of job stress were significantly correlated with hypochondria. Indeed, hypochondria caused by corona reduces a person's physical and mental capacity to perform their tasks, which significantly increases job stress. The relationship between hypochondria caused by corona and occupational stress has not been studied in previous studies, but studies have been conducted on the relationship between stress and hypochondria caused by corona. Gong's study showed that COVID19 caused hypochondria and increased stress in people with the disorder, which increased the demand for medical services (Gong et al., 2020). Past studies have also shown that the prevalence of COVID19 and the prevalence of past diseases such as SARS and H1N1 caused the general population to experience different levels of stress (Aghili and Arbabi, 2020). China's findings show that more than 8.1% of the people experienced average to severe stress level caused by the outbreak of coronavirus (Wang et al., 2020a). Among the dimensions of job stress, responsibility showed the greatest correlation with hypochondria. It is clear that hypochondria and fear of corona make people more cautious in their care to prevent the disease and maintain their health. In other words, they have a greater sense of responsibility for their own and their family's health than their responsibility for their job. The present study showed that there is a negative correlation between job stress and resilience, which means that resilience diminishes with increasing stress, and vice versa, with increasing resilience, job stress also declines. According to a study by Wu and Santarone, there was a positive role for resilience in reducing stress and other mental disorders such as disease anxiety and COVID19-related fear (Santarone et al., 2020; Wu et al., 2020).

### 4.3 Path analysis

The results of path analysis indicated that a person with hypochondria himself seems to be able to reduce productivity through two paths. Initially, hypochondria reduces productivity by increasing job stress, and in the second way, hypochondria itself reduces productivity by reducing resilience. Previous studies have examined the relationship between job stress and resilience and productivity. Hoboubi et al. investigated the effect of job stress on productivity of 125 workers working in one of the petrochemical industries of Iran. The data was collected using SPIO job stress questionnaire and Hersi Gold Smith Productivity. The results showed that there was no significant positive relationship between all aspects of job stress and employee well-being. Also, among the six dimensions of job stress, two dimensions of inadequacy and role ambiguity have the greatest impact on people's productivity. Workers should take preventive and supportive measures to improve job stress, especially in the two dimensions of inadequacy and role ambiguity (Hoboubi et al., 2017). Bharathi et al. also studied the relationship between stress and productivity among 92 women employees in the IT sector. The results of the correlation test between job stress and productivity showed that with increasing job stress, the productivity of female employees in the IT sector diminished (Bharathi and Gupta, 2017). Studies in various occupations such as administrative work, treatment personnel, and textile industry have pointed to the inverse relationship between job stress and productivity, which is consistent with the results of the present study (Alam, 2016; Ramos-Galarza and Acosta-Rodas, 2019; Sallon et al., 2017). Among studies in which the relationship between resilience and productivity has been mentioned, Wang c study can be referred. This study stated that the prevalence of COVID19 virus is a very important challenge for cognitive resilience (Wang et al., 2020a). Kobasa said that people with higher cognitive resilience are less likely to become ill and are healthier than others, despite experiencing stressful events throughout their lives. As a result, this issue can be effective in increasing the productivity of these people (Kobasa, 1979). In another study, Inzlicht et al. found that resilient individuals can overcome a variety of adverse effects as well as physical and emotional fatigue from work, and maintain their mental health. As a result, it increases people's ability to cope with work-related stress and enhances productivity (Inzlicht et al., 2006).

Overall, based on the results of studies on the effect of stress on productivity and the impact of resilience on productivity as well as the influence of resilience on stress or vice versa, it can be stated that two important pathways of hypochondria caused by coronavirus on workers' productivity have been investigated in the presented model. Although other variables may modify the effect of hypochondria on productivity, in the model proposed in this study, only two variables of job stress and resilience were considered as modifiers of hypochondria effect on productivity, which can be considered as one of the limitations of this study. Another limitation of this study was that all participants were male and the effect of hypochondria was not studied on the female workers' productivity. Also, the workers occupied in the central workshop were only investigated and the workers of other industrial parts can be studied in the next research. Moreover, the researchers could not collect some data through face-to-face interview because of conditions of COVID 19 disease.

## 5 Conclusion

The results of the study generally suggested that coronavirus disease has caused the spread of hypochondria mental disorder. In the present study, hypochondria could reduce the productivity of workers through two ways of increasing job stress and reducing workers' resilience. Thus, in order to mitigate the effect of hypochondria on productivity, it is recommended to focus on this important factor. Also, preventive and control measures to reduce job stress and enhance workers' resilience should be considered by managers and employers of various industries so that they can improve employees' current productivity and mental health in this critical situation. Moreover, the managers must try to decrease the hypochondria due to COVID 19 through increased awareness of personnel on this disease and the preventive measures and encouraging the workers to perform the hygiene protocols.

## Declarations

### Author contribution statement

Saeid Yazdanirad: Conceived and designed the experiments; Analyzed and interpreted the data; Wrote the paper.

Marziyeh Sadeghian: Conceived and designed the experiments; Analyzed and interpreted the data.

Mahsa Jahadi Naeini and Sayed Mahdi Mousavi: Performed the experiments; Wrote the paper.

Milad Abbasi: Contributed reagents, materials, analysis tools or data; Wrote the paper.

### Funding statement

This research did not receive any specific grant from funding agencies in the public, commercial, or not-for-profit sectors.

### Data availability statement

The authors do not have permission to share data.

### Declaration of interests statement

The authors declare no conflict of interest.

### Additional information

No additional information is available for this paper.
